# Simulation-based medical education for Ambulance Jet and Helicopter Emergency Medical Services: A program description and evaluation

**DOI:** 10.15694/mep.2021.000145.1

**Published:** 2021-05-25

**Authors:** Sabine Nabecker, Rafaela Pfeffer, Stefan Lötscher, Yves Balmer, Lorenz Theiler, Robert Greif, Roland Albrecht

**Affiliations:** 1 Department of Anaesthesiology and Pain Medicine; 2 Department of Anaesthesiology and Pain Medicine; 3 Department of Anaesthesia; 4 Bern Simulation and CPR-Centre; 5 Department of Anaesthesia; 6 Department of Anaesthesiology and Pain Medicine; 7 Swiss Air-Rescue Rega

**Keywords:** simulation, Emergency Medical Services, non-technical skills, human factors, simulation-based medical education

## Abstract

This article was migrated. The article was marked as recommended.

**Introduction:** In aviation, crew resource management trainings are established methods to enhance safety, a method that also gained popularity in medicine. In 2015, the Swiss Air Rescue (Rega) Helicopter Emergency Medical Services decided to start a simulation-based medical education program for its helicopter and ambulance jet crews (emergency physicians, paramedics/flight nurses and pilots). The aim of this program was to improve technical skills and the application of human factors during rescue missions. This report shows a five-year summary of the participants’ course evaluation.

**Methods:** A 1-day high-fidelity simulation on crisis resource management with video-assisted debriefing took place at 3 centres, two in Switzerland; one in Germany. Crew members participated once per year. Simulation covered critical situations in the helicopter or jet, during handovers at an intensive care unit or in ambulances. Extra Corporeal Membrane Oxygenation and Intra-Aortic Balloon Pump use was simulated during helicopter transports. Additionally, four times per year flight crews rehearsed basic and advanced life support skills using low-fidelity equipment between missions. Participants answered an anonymized course evaluation survey. Answers were rated on a Numeric Rating Scale ranging from 1=no agreement to 5=total agreement.

**Results:** 329 participated and answered the questionnaire; 50% were emergency physicians, 40% paramedics, 9% flight nurses, and 1% pilots. Participants agreed that the course taught competencies that were useful for their clinical practice. However, confidence to apply Extra Corporeal Membrane Oxygenation or Intra-Aortic Balloon Pump skills was significantly lower compared to other emergency competencies. Instructors were rated as experienced, engaged and motivated, as well as responsive to course participants.

**Conclusions:** This simulation-based medical education program, with the goal to increase patient’s safety and outcome, was launched successfully. Participants especially valued the time to reflect on clinical performance as well as on crew interaction and ways to apply human factors to improve their team performance and task management.

## Introduction

Health care professionals benefit from simulation-based medical education ( McLaughlin *et al.*, 2008). Simulation-based medical education uses environments and scenarios that mimic the real-life clinical situations and experiences as realistically as possible ( Cheng *et al.*, 2007). Simulation-based medical education allows acquisition of knowledge, and training of technical and dynamically evolving procedural skills, invasive interventions or handling of complex interactions in high-hazard environments without real patient interaction ( Gaba, 2004). Therefore, simulation-based medical education allows health care professionals to apply technical and non-technical skills without putting themselves and patients at risk ( Cheng *et al.*, 2007; Dotson *et al.*, 2018). Additionally, the main purpose of applying simulation-based medical education is the proper handling of human factors during crisis situations. Studies have shown improvements of non-technical skills due to simulation-based medical education ( McCulloch *et al.*, 2009). Other studies have shown poor human factors application during medical care affect patient management and outcome ( Mishra *et al.*, 2008; Gjeraa *et al.*, 2016).

In 2011 McGaghie *et al.* demonstrated in a meta-analysis the superiority of simulation-based medical education over the traditional clinical education ( McGaghie *et al.*, 2011). The majority of published simulation studies involved to some extent team-training ( Weaver, Dy and Rosen, 2014). Another Swiss simulation program for Helicopter Emergency Medical Services staff, with a main focus on mountain rescue, showed that simulation-based education subjectively improved participants’ human factor skills application ( Pietsch *et al.*, 2016). Helicopter Emergency Medical Services crews’ simulation in Norway included non-technical skills, like decision-making, leadership, communication, situational awareness, teamwork, but also management of stress and coping with fatigue ( Rasmussen *et al.*, 2019). Teamwork in a highly demanding emergency medicine setting often involves rapid spontaneous team formation, as individual specialists are often unfamiliar with one another and have limited experience in working together as a team of professionals ( Weaver, Dy and Rosen, 2014).

The Swiss Air Rescue (Rega), the biggest Helicopter Emergency Medical Service in Switzerland, has 18 helicopters and 3 ambulance jets on 12 helicopter bases as well as the airport in Zürich. It is a non-profit organization that has performed, in 2019 alone, 16’782 missions ( Swiss Air Rescue, Rega, 2020a). Helicopter crews consist of an emergency physician, a paramedic and a pilot; while ambulance jet crews include an emergency physician, an intensive care nurse and two jet pilots ( Swiss Air Rescue, Rega, 2020b).

This paper reports the setup of a simulation-based medical education program for an internationally operating air-borne Emergency Medical Service, a description of the simulation cases used for this program, and the result of the evaluation survey completed by all participants between 2015 and 2019.

## Methods

This project, being purely observational, does not fall under the Swiss Human Research Act as declared by the Bern Cantonal Ethics Committee, in Bern, Switzerland (Req-217-00180; 16.03.2017). The survey was performed between 2015 and 2019 at the Department of Anaesthesiology and Pain Medicine, at Bern University Hospital, Switzerland.

All crew members (emergency physicians, paramedics, flight nurses and pilots) of the Swiss Air Rescue (Rega) were invited to take part in this mandatory simulation program at least once per year. All participants of this Rega simulation program took part in the survey directly after the completion of their respective simulation. Participants provided written informed consent to the publication of this data.

### The Rega simulation-based medical education program

This simulation program was organized at 3 different centres: (i) Bern Simulation- and CPR Centre (BeSiC) at the Bern University Hospital, Switzerland; (ii) Swiss Institute for Emergency Medicine (SIRMED) in Nottwil, Switzerland; and (iii) the Maquet Extra Corporeal Membrane Oxygenation (ECMO) and Intra-Aortic Balloon Pump (IABP) training centre in Rastatt, Germany.

The two Swiss simulation centres offered a 1-day crisis resource management simulation using high-fidelity simulation manikins. It was followed by a video-assisted debriefing led by experienced, trained and certified simulation instructors. The goal was to help enable deeper reflexive learning that results in an action plan leading to improved future crew performance. Crisis resource management includes among others: knowing your environment; anticipation, sharing and review of a plan; clear leadership and role clarity; effective communication; calling for help early; considerate allocation of equipment and personnel; avoidance of fixation errors; distribution of workload and support of the team ( Carne, Kennedy and Gray, 2012). The simulation focused on highly complex medical emergency situations happening during an air rescue, during a hand-over from paramedics or during a hand-over while transferring a patient between hospitals. The aim of this simulation program was to reflect on the influence of human factors on individual and team performance during life-threatening medical emergencies. This program focused on individuals’ communication structure, team leadership and team membership, as well as concise dynamic decision-making skills and situational awareness under time pressure and stress.

The 1-day simulation-based medical education curricula consisted of a half-day full-scale simulation and a half-day of skills tutorials. Skills tutorials composed of thoracic drainage insertion on pig hemi-thoraces, intraosseous access on pig and training bones, and emergency front of neck access on pig larynges covered with pig skin. There was also the opportunity to practice ventilation strategies on a specific simulator (TestChest; Organis, Landquart, Switzerland) connected to the transportable ventilator of the respective emergency team.

After familiarization with the equipment and the full-scale simulator manikin, Rega teams were immersed in emergency scenarios. For example, one scenario was an urgent patient transfer request from an intensive care unit to a higher-level hospital. In the radio-report the patient was stable, however, when the crews entered the simulation scenario, the patient showed signs of an active internal bleeding and was unstable. The learning goal was to effectively communicate during the emergency handover; specifically, to identify the instability and request for urgent surgical stabilisation before transfer.

Another example was a pre-hospital scenario, the helicopter crew was ordered to rescue a patient who fell off a scaffolding. On arrival at the scene, parts of the scaffolding were on the ground. The patient’s trachea had already been intubated by an ambulance crew just prior to the arrival of the Rega crew. During handover, the paramedic of the ambulance crew claimed that he had intubated the patient correctly, but that the end-tidal carbon dioxide probe was not working. The first objective of this scenario was to identify the need for safety first in a potentially unsafe environment. The second objective was to quickly detect the oesophageal intubation, correct the mistake while applying cervical spine protection but also making sure to receive handover from the first crew.

In the German ECMO and IABP training centre, a 1-day simulation of critical incidents in ECMO and IABP use during a helicopter transport mission was offered.

In addition to the high-fidelity simulations, all participants of the simulation program were encouraged to rehearse and practise knowledge and skills of basic and advanced life support at least four times per year at their helicopter bases between missions. For this, low-fidelity equipment and manikins were used. These on-site practise rehearsals gave the local teams the chance to apply learned competencies on pre-defined standardized clinical situations ( Miller *et al.*, 2016). These 30-min rehearsals were designed to fit into short time slots between outreach missions.

### Measurements

Participants were asked to provide the following information at the end of the simulation: setting of simulation (helicopter, ambulance jet, ECMO/IABP), profession (emergency physician, paramedic, flight nurse, pilot), primary base (helicopter mountain or midland base, ambulance jet base), and centre (BeSiC, Sirmed, Maquet).

Participants were asked to rate the following course parameters on a Numeric Rating Scale ranging from 1=no agreement to 5=total agreement: subjective impression of the course; the course content was hands-on; “I am confident to use the course content if necessary”; if they would recommend the course to others; expertise, engagement, and motivation of instructors; ability of instructors to be responsive to course participants; general mood of the group; course organization as expected; quality of educational materials, infrastructure and ambiance; as well as the course content (simulation sessions and skills trainings).

### Statistics

The convenience sample consists of all participants of this Rega simulation program between 2015 and 2019. Due to the exploratory character of this study no sample size calculation was performed.

Stata version 16.0 (StataCorp LT, Texas, USA) was used for statistical analysis. Descriptive statistics was used to summarize the answers from the course evaluation. Kruskal-Wallis test compared the answers from the questionnaire between the different test settings (helicopter, jet, ECMO/IABP). Mann-Whitney-U test was used for post-hoc pairwise comparison. Data are presented as number (percentage) or mean ± standard deviation. A p-value of < 0.05 was considered statistically significant.

## Results/Analysis

All 329 Rega simulation participants returned the survey and were included in this report. Three different simulation settings were included: helicopter simulation 239 (73%) participants, ambulance jet simulation 55 (23%) participants, and ECMO/IABP simulation 35 (15%) participants.

Participants’ professions were: 165 (50%) emergency physicians, 131 (40%) paramedics, 31 (9%) flight nurses, and 2 (1%) pilots. One hundred thirty-seven (42%) participants reported to be primarily based at a helicopter mountain base, 109 (33%) at a helicopter midland base and 27 (8%) at an ambulance jet base.

The simulations took place at the Bern Simulation and CPR-Centre for 228 (69%) participants, for 66 (20%) at the Sirmed educational centre and for 35 (11%) at the Maquet training centre.

Table 1 shows the answers to the course evaluation questionnaire. All evaluation questions except one were rated comparably between training centres ( Table 1). Participants of the ECMO/IABP simulation rated the answer to the statement: “I am confident to use the course content if necessary” significantly lower compared to both helicopter and jet crews (both p < 0.001).

All skills training sessions were rated higher in the jet group than in both other groups (all p-values in post-hoc analysis < 0.01).

**Table 1:** Answers to the course evaluation

Parameter	helicopter n=239	ambulance jet n=55	ECMO/IABP n= 35	p-value
**Overall**				
Personal impression of the course	4.8 ± 0.4	4.9 +/- 0.3	4.9 +/- 0.3	0.177
The course content was hands-on	4.8 ± 0.4	4.9 +/- 0.3	4.8 +/- 0.5	0.300
I am confident to use the course content if necessary	4.6 ± 0.5	4.7 +/- 0.4	3.8 +/- 0.7	< 0.001 ^A^
I recommend the course to other health care professionals	4.8 ± 0.4	5.0 +/- 0.2	4.8 +/- 0.2	0.068
**Rating of instructors’**				
expertise	4.9 ± 0.3	5.0 ± 0.0	4.9 ± 0.4	0.501
engagement and motivation	4.9 ± 0.2	5.0 ± 0.2	4.9 ± 0.2	0.854
ability to be responsive to course participants	4.8 ± 0.4	5.0 ± 0.1	4.9 ± 0.3	0.179
General mood of the group	4.8 ± 0.4	5.0 ± 0.0	4.9 ± 0.4	0.194
**Course organisation**				
The organisation corresponded to my expectations.	4.8 ± 0.4	4.9 ± 0.2	4.9 ± 0.4	0.204
Quality of educational material	4.7 ± 0.5	4.9 ± 0.3	4.8 ± 0.6	0.170
Infrastructure and ambiance	4.8 ± 0.4	4.9 ± 0.4	5.0 ± 0.0	0.053
**Course content**				
Simulation session 1	4.6 ± 0.5	4.8 ± 0.5	4.8 ± 0.5	0.170
Simulation session 2	4.7 ± 0.5	4.9 ± 0.4	4.8 ± 0.5	0.041
Simulation session 3	4.8 ± 0.4	4.9 ± 0.3	4.2 ± 0.7	< 0.001 ^B^
Skills training 1	4.5 ± 0.6	4.8 ± 0.4	3.4 ± 1.1	< 0.001 ^C^
Skills training 2	4.4 ± 0.7	4.8 ± 0.4	4.4 ± 0.7	0.008 ^D^

Numeric Rating Scale (NRS 1-5; 1=no agreement; 5=total agreement).

Data are mean ± standard deviation.

Extra Corporeal Membrane Oxygenation (ECMO), Intra-Aortic Balloon Pump (IABP).

^A^ post-hoc analysis revealed significant differences between helicopter and ECMO/IABP and between ambulance jet and ECMO/IABP (all p < 0.01), no correction factor applied.

^B^ post-hoc analysis revealed significant differences between helicopter and ambulance jet, between helicopter and ECMO/IABP and between ambulance jet and ECMO/IABP (all p < 0.01), no correction factor applied.

^C^ post-hoc analysis revealed significant differences between helicopter and ambulance jet, between helicopter and ECMO/IABP and between ambulance jet and ECMO/IABP (all p < 0.01), no correction factor applied.

^D^ post-hoc analysis revealed significant differences between helicopter and ambulance jet, and between ambulance jet and ECMO/IABP (all p < 0.01), no correction factor applied.

## Discussion

This paper reports the development of a simulation-based medical education program for the Swiss Air Rescue (Rega) and a five-year summary of the participants’ course evaluation. The curriculum was well received and rated as highly applicable to the clinical practice of the helicopter rescue as well as for the ambulance jet crews. The high-fidelity simulation as well as the hands-on skills tutorials were rated as highly positive and the instructors of the teaching centres were found to be engaged, motivated and competent. All participants stated they would recommend the program to their colleagues. In the ECMO/IABP setting, the data showed participants were less confident to use the course content than in any other settings.

All participants agreed that the simulation-based medical curriculum was useful for their clinical practice. These findings are consistent with other studies who found mostly positive reactions to team-training interventions, which showed over 80% positive rating on the practical application of their simulation experience ( Weaver, Dy and Rosen, 2014). Reid *et al.* reported a comparable rate of agreement that the simulation training was relevant to their participant’s work ( Reid *et al.*, 2012).

The Rega simulation participants rated their self-efficacy in being confident to use the learned competencies in daily rescue missions as high, however, participants from ECMO/IABP training rated it significantly lower than participants from the helicopter and ambulance jet simulations. Another study reported a significant increase in self-efficacy taken immediately after the team training and taken three months later ( Weaver, Dy and Rosen, 2014). The possible contributing factors for a comparatively lower rating in the ECMO/IABP group may be in the unfamiliarity with the devices used, or that such ECMO/IABP transports are rarely necessary. Therefore, physicians and paramedics would not have been used to troubleshooting these devices, hence they may have felt overall reluctance. In real life situations usually a perfusion specialist from the local ECMO centre would have accompanied the ECMO/IABP transport. Doing this survey, identified this issue and the ECMO/IABP simulation was improved by adding such perfusion specialists for the future.

Participants of another simulation-based mountain Helicopter Emergency Medical Services educational program reported a subjective increase in the participants’ structured decision-making skills and increased certainty in professionally managing emergency situations ( Pietsch *et al.*, 2016). However, it should be noted that this simulation program taught a lot about technical skills, whereas in the Rega simulation curriculum, the focus was on crisis resource management, and included mountain and midland helicopter bases crews, as well as the ambulance jet crews.

In many situations, cost-effectiveness may be the limitation to simulation trainings. However, in 2018 Dotson *et al.* showed that the average cost per participant was lower in a group of nurses/paramedics trained with simulation, compared to without ( Dotson *et al.*, 2018). Although this difference was not significant, the report shows that simulation programs might have a role in saving money in the long run when training airborne Emergency Medical Services crew members.

A narrative synthesis revealed that team-training enhanced positive attitudes and teamwork, as well as improve clinical knowledge of participants which led to better clinical care and patient outcomes ( Weaver, Dy and Rosen, 2014). Patterson *et al.* showed that simulation-based training improved patient safety in paediatric emergency departments ( Patterson *et al.*, 2013). Most randomized controlled trials in simulation research assess the educational intervention rather than the patient outcome ( Chauvin *et al.*, 2018), the assumption is that the positive effect of simulation on team performance benefits patient care and ultimately will benefit patient outcome. This needs to be further investigated in future studies.

One limitation in the Rega simulation program is that it was only offered to the crew members of the Swiss Air Rescue (Rega) in Switzerland. Other Emergency Medical Services organization members or organizations in different countries might obtain different results. Also, this report only showed the results of a subjective course evaluation by course participants. Future investigations need to establish evidence to verify whether such a simulation program has an effect on patient outcome.

Another limitation is in the lack of demographic data from the course participants. The survey was kept anonymous to ensure high return rate, and to guarantee absolute privacy for teams working closely together.

On the other hand, a strength to this study is in the 100% return rate of the course evaluation forms, as well as in combining different educational approaches like high-fidelity crisis resource management simulation with low-fidelity skills training. Educational variety engages adult learners better and improves learning outcomes ( Miller *et al.*, 2016).

## Conclusion

In conclusion, the implementation of the mandatory simulation-based medical education program of the Swiss Air Rescue (Rega) was highly valued by over 300 participants (emergency physicians, paramedics, flight nurses, pilots) from the helicopter and ambulance jet crews. Participants of the simulation program valued its benefits and its relevance to their clinical practice; and rated the course organizers’ and instructors’ competencies highly. Further investigation is needed to validate whether human factor education in Emergency Medical Services teams enhances patient outcome.

## Take Home Messages

• This report shows the successful launch of a simulation-based medical education program for helicopter and ambulance jet crews.
• Participants valued the time to reflect on clinical performance, on crew interaction and ways to apply human factors to improve their team performance and task management.
• Further investigation is needed to validate whether human factor education in Emergency Medical Services teams enhances patient outcome.

## Notes On Contributors

**Sabine Nabecker** is currently a clinical fellow for education and simulation in anesthesia at the Department of Anesthesia and Pain Management, at the Sinai Health System, University of Toronto. Apart form that she is also a staff anesthesiologist at the Department of Anaesthesiology and Pain Medicine, Bern University Hospital, University of Bern, Bern, Switzerland.

**Rafaela Pfeffer** is a resident in anesthesia and a doctoral thesis student at the Department of Anaesthesiology and Pain Medicine, Bern University Hospital, University of Bern, Bern, Switzerland.

**Stefan Lötscher** is chair of the Department of Anaesthesia, Kantonsspital Uri, Altdorf, Switzerland.

**Yves Balmer** is chair of the Bern Simulation and CPR-Centre, Department of Anaesthesiology and Pain Medicine, Bern University Hospital, University of Bern, Bern, Switzerland.

**Lorenz Theiler** is a professor of anesthesia and chair of the Department of Anaesthesia, Kantonsspital Aarau, Aarau, Switzerland.

**Robert Greif** is a professor of anesthesia and senior consultant at the Department of Anaesthesiology and Pain Medicine, Bern University Hospital, University of Bern, Bern, Switzerland; he is also professor of Medical Education at the School of Medicine, Sigmund Freud University Vienna, Vienna, Austria.

**Roland Albrecht** is medical chair of the Swiss Air-Rescue Rega, Zurich, Switzerland.

## Declarations

Robert Greif is the current Board Director of Education and Training of the European Resuscitation Council and Chair of the ILCOR Task Force on Education, Implementation and Team. Sabine Nabecker is the current Education Representative of the ‘Young ERC’ of the European Resuscitation Council. The other authors report no conflict of interest.

## References

[ref1] Carne B. Kennedy M. and Gray T. (2012) Review article: Crisis resource management in emergency medicine. Emerg Med Australas. 24(1), pp.7–13. 10.1111/j.1742-6723.2011.01495.x 22313554

[ref2] Chauvin A. Truchot J. Bafeta A. Pateron D. (2018) Randomized controlled trials of simulation-based interventions in Emergency Medicine: a methodological review. Intern Emerg Med. 13(3), pp.433–444. 10.1007/s11739-017-1770-1 29147942

[ref3] Cheng A. Duff J. Grant E. Kissoon N. (2007) Simulation in paediatrics: An educational revolution. Paediatr Child Health. 12(6), pp.465–468. 10.1093/pch/12.6.465 19030409 PMC2528751

[ref4] Dotson M. P. Gustafson M. L. Tager A. and Peterson L. M. (2018) Air Medical Simulation Training: A Retrospective Review of Cost and Effectiveness. Air Med J. 37(2), pp.131–137. 10.1016/j.amj.2017.11.012 29478579

[ref5] Gaba D. M. (2004) The future vision of simulation in health care. Qual Saf Health Care. 13Suppl1, pp.i2–10. 10.1136/qhc.13.suppl_1.i2 15465951 PMC1765792

[ref6] Gjeraa K. Jepsen R. M. Rewers M. Ostergaard D. (2016) Exploring the relationship between anaesthesiologists’ non-technical and technical skills. Acta Anaesthesiol Scand. 60(1), pp.36–47. 10.1111/aas.12598 26272742

[ref7] McCulloch P. Mishra A. Handa A. Dale T. (2009) The effects of aviation-style non-technical skills training on technical performance and outcome in the operating theatre. Qual Saf Health Care. 18(2), pp.109–115. 10.1136/qshc.2008.032045 19342524

[ref8] McGaghie W. C. Issenberg S. B. Cohen E. R. , Barsuk, J. H., (2011) Does simulation-based medical education with deliberate practice yield better results than traditional clinical education? A meta-analytic comparative review of the evidence. Acad Med. 86(6), pp.706–711. 10.1097/ACM.0b013e318217e119 21512370 PMC3102783

[ref9] McLaughlin S. Fitch M. T. Goyal D. G. Hayden E. (2008) Simulation in graduate medical education 2008: a review for emergency medicine. Acad Emerg Med. 15(11), pp.1117–1129. 10.1111/j.1553-2712.2008.00188.x 18638028

[ref10] Miller J. O. Thammasitboon S. Hsu D. C. Shah M. I. (2016) Continuing Medical Education for Air Medical Providers: The Successes and Challenges. Pediatr Emerg Care. 32(2), pp.87–92. 10.1097/PEC.0000000000000416 26841111

[ref11] Mishra A. Catchpole K. Dale T. and McCulloch P. (2008) The influence of non-technical performance on technical outcome in laparoscopic cholecystectomy. Surg Endosc. 22(1), pp.68–73. 10.1007/s00464-007-9346-1 17479324

[ref12] Patterson M. D. Geis G. L. LeMaster T. and Wears R. L. (2013) Impact of multidisciplinary simulation-based training on patient safety in a paediatric emergency department. BMJ Qual Saf. 22(5), pp.383–393. 10.1136/bmjqs-2012-000951 23258388

[ref13] Pietsch U. Knapp J. Ney L. Berner A. (2016) Simulation-Based Training in Mountain Helicopter Emergency Medical Service: A Multidisciplinary Team Training Concept. Air Med J. 35(5), pp.301–304. 10.1016/j.amj.2016.05.006 27637441

[ref14] Rasmussen K. Langdalen H. Sollid S. J. M. Abrahamsen E. B. (2019) Training and assessment of non-technical skills in Norwegian helicopter emergency services: a cross-sectional and longitudinal study. Scand J Trauma Resusc Emerg Med. 27(1), p.1. 10.1186/s13049-018-0583-1 30616604 PMC6323750

[ref15] Reid J. Stone K. Brown J. Caglar D. (2012) The Simulation Team Assessment Tool (STAT): development, reliability and validation. Resuscitation. 83(7), pp.879–886. 10.1016/j.resuscitation.2011.12.012 22198422

[ref16] Swiss Air Rescue, Rega, (2020a): Swiss Air Rescue, Rega. This is how we help you. Available at: https://www.rega.ch/en/our-missions/this-is-how-we-help-you( Accessed: 28/10/2020).

[ref17] Swiss Air Rescue, Rega, (2020b): Swiss Air Rescue, Rega. This is how we help in Switzerland. Available at: https://www.rega.ch/en/our-missions/this-is-how-we-help-you#in-switzerland( Accessed: 28/10/2020).

[ref18] Weaver S. J. Dy S. M. and Rosen M. A. (2014) Team-training in healthcare: a narrative synthesis of the literature. BMJ Qual Saf. 23(5), pp.359–372. 10.1136/bmjqs-2013-001848 PMC3995248 24501181

