# Supplementary material for: Simulation-based medical education for Ambulance Jet and Helicopter Emergency Medical Services: A program description and evaluation
Source: MedEdPublish (2016). 2021 May 25;10:145. [Version 1] doi: 10.15694/mep.2021.000145.1 (PMC10939555; doi:10.15694/mep.2021.000145.1)
Supplement: Peer review history

# Reviewer report: Reviewer response for version 1

**DOI:** 10.21956/mep.19107.r27555

**Published:** 2021-10-03

**Authors:** David Bruce

**Affiliations:** 1 None

This review has been migrated. The reviewer awarded 4 stars out of 5 I enjoyed reading this is interesting and informative paper. The authors describe and evaluate the simulation-based training programme provided by the Swiss Air Rescue (Rega) service, which completes just under 17,000 missions per year. The training is aimed at the critically ill patient and has a human factors approach looking at technical and non-technical skills. The training is provided at two sites in Switzerland, which provides tutorials, technical skills training and scenarios that involve team using their full range of technical and non-technical skills. A third training centre in Germany offer simulation in ECMO and IABP. In addition, basic and advanced life support skills training took place four times per year in more local centres. All participants in the training programme from 2015 to 2019 took part in the evaluation – a questionnaire that considered course content, materials, satisfaction with instructions received and confidence in using the course content. The results are clearly presented with straightforward descriptive statistics. The discussion is well structured and looks at the importance of human factors in the delivery of critical care by teams who may be unfamiliar with each other. A well written paper. As a non-expert reader, I wondered if the training provided at the German site was perhaps different to the two Swiss sites – and ideally would have liked some more information about any differences. I would have liked to hear how this training had impacted the care they provided such as what aspects of the training had perhaps altered their own and the teams approach to a particular patient’s care – but perhaps this a future paper. Although this study is based on the Swiss Air Rescue, I think it has positive messages for all involved in team delivery of complex care.

Reviewer Expertise:

NA

No decision status is available

# Reviewer report: Reviewer response for version 1

**DOI:** 10.21956/mep.19107.r27553

**Published:** 2021-07-16

**Authors:** Lars Egger

This review has been migrated. The reviewer awarded 4 stars out of 5 This observational study addresses an important topic “crew resource management trainings” among EMS helicopter crews. The main result focusses on feasibility of CRM trainings and general self-evaluation and acceptance of the training by the participants. As the authors note, further studies focusing on patient safety more specifically are necessary. Of interest within this study also would be the correlation of participants performance rated by the investigators towards participants self-assessment.

Reviewer Expertise:

NA

No decision status is available

# Reviewer report: Reviewer response for version 1

**DOI:** 10.21956/mep.19107.r27552

**Published:** 2021-07-15

**Authors:** Kurt Ruetzler

This review has been migrated. The reviewer awarded 4 stars out of 5 This is a well written manuscript about safety in aviation, specifically in 2 separate HEMS systems in Switzerland and Germany. The authors performed 1-day high-fidelity simulations on crisis resource management at 3 separate centers. The manuscript summarizes the course evaluation by 329 participants over 5 year time period. The methodology is clearly written and comprehensible. The results are clearly stated. The conclusion is straight forward and is supported by the data obtained by this study. This manuscript is clearly relevant to all colleagues involved in aviation/ HEMS and emergency medicine in general. Well done!

Reviewer Expertise:

NA

No decision status is available

# Reviewer report: Reviewer response for version 1

**DOI:** 10.21956/mep.19107.r27554

**Published:** 2021-07-13

**Authors:** Richard Hays

**Affiliations:** 1 James Cook University

This review has been migrated. The reviewer awarded 4 stars out of 5 This article is well written and describes well how a national advanced medical retrieval service approached training staff in high level. critical care skills in three locations using high fidelity simulation. The training program appears to have been well-designed and able to be implemented on a large scale. The participant evaluation (low level but 100% response rate!) was positive. This should encourage further training programs like this to be conducted in other similar services. The relevance of this article is mostly with those engaged in high level simulation of advanced postgraduate skills, but the principles are similar in all simulation settings.

Reviewer Expertise:

NA

No decision status is available
